# Challenge or Threat? The Effects of the Standard and a Second-Generation Mindfulness Intervention with Buddhist Practices on Cognitive Appraisals of Stress: Secondary Analysis of a Randomized Controlled Experiment Performed in Switzerland

**DOI:** 10.1007/s10943-023-01964-8

**Published:** 2023-12-22

**Authors:** Liudmila Gamaiunova, Pierre-Yves Brandt, Matthias Kliegel

**Affiliations:** 1 Institute for Social Sciences of Religions (ISSR), University of Lausanne, Quartier UNIL-Chamberonne, 1015 Lausanne, Switzerland; 2 Swiss National Center of Competences in Research LIVES–Overcoming Vulnerability: Life Course Perspectives, Lausanne, Switzerland; 3 Institute of Psychology, University of Lausanne, 1015 Lausanne, Switzerland

**Keywords:** Buddhist meditation, Mindfulness, Contemplative practice, Stress, Cardiovascular response, Cognitive appraisals, Challenge and threat, TSST

## Abstract

Contemplative approaches rooted in Buddhist traditions have been linked to the attenuation of response to social stress. Anticipatory cognitive appraisals of social situations potentially represent a mechanism explaining the stress-reducing effects of contemplative practices. The cognitive appraisal of threat is associated with an anticipated loss of social self-esteem. In contrast, the cognitive appraisal of challenge involves recognizing the potential for gain or growth in stressful situations and is associated with a more adaptive cardiovascular response. In this secondary analysis of a randomized controlled experiment performed in Switzerland, we evaluated the effects of two contemplative interventions on cognitive appraisals of challenge and threat and associated physiological profiles. The interventions were a standard Mindfulness-Based Stress Reduction (MBSR) program and a new program (MBSR-B), which included several elements from Buddhist practices. After an eight-week intervention, participants completed the Trier Social Stress Test (TSST) and underwent the assessment of primary cognitive appraisals and cardiovascular response to stress. The results demonstrated greater challenge appraisal in the MBSR (*n* = 20) and MBSR-B (*n* = 21) groups compared to Control (*n* = 24), and MBSR-B participants scored higher on the challenge than threat appraisal. At the physiological level, the groups did not differ on changes in cardiac output and total peripheral resistance. Still, an exploratory analysis demonstrated that the MBSR-B group’s cardiovascular profile best resembled challenge appraisal. The results suggest that contemplative approaches foster challenge appraisal, contributing to a more adaptive response to stress.

**Supplementary Information:**

The online version contains supplementary material available at 10.1007/s10943-023-01964-8.

## Introduction

The detrimental effects of stress on health are well documented (O'Connor et al., 2021). Psychological stressors, especially of a social nature, evoke robust activation of different physiological systems, often exceeding current somatic and metabolic demands. In terms of pathophysiology, an exaggerated stress response strains the cardiovascular system (Obrist, 2012). It can lead to allostatic load: “the wear and tear on the body,” resulting from heightened neural or neuroendocrine response (McEwen, 1998). The cognitive interpretation of a stressor largely determines the amplitude of the psychological strain. According to the Transactional Model of Stress and Coping (Folkman & Lazarus, 1984), stress is born in an individual’s interaction with an environment and mainly depends on the appraisal of a situation. The cognitive appraisal is constructed based on a judgment about the implications of a situation (primary appraisal) and the ability to cope with it (secondary appraisal). The initial evaluation of a situation constitutes the primary appraisal and results in the experience of threat, challenge, or harm in the case of a stressful event. The primary appraisal will likely determine the magnitude of the psychophysiological stress response. However, not only the magnitude of the stress response but also the particularity of physiological activation (arousal in response to stress) has important implications. According to the biopsychosocial (BPS) model of challenge and threat (Tomaka et al., 1993), cognitive evaluation of a stressor results in a specific physiological profile, where challenge represents a more adaptive response to psychological stress than threat.

Mindfulness-based interventions (MBIs)—behavioral approaches rooted in Buddhist practices, such as mindfulness meditation—have been linked to stress attenuation in the context of social stressors (Morton et al., 2020). Since stress response is highly related to the anticipatory cognitive appraisal—an evaluation that happens before a situation—it represents a potential mechanism linking mindfulness-based approaches and stress attenuation. The above-mentioned cognitive mechanisms might be particularly pronounced in meditation programs aimed at the development of cognitive insight, purpose, and meaning (Dahl & Davidson, 2019) or the so-called second-generation mindfulness-based interventions (SG-MBIs) (Van Gordon & Shonin, 2020). In addition to mindfulness, these practices engage larger Buddhist practice frameworks and a broader set of traditional techniques. In addition to stress-protecting mechanisms brought about by the cultivation of mindfulness (Creswell & Lindsay, 2014; Vago & David, 2012), SG-MBIs have the potential to engage in religious or spiritual coping, as specific beliefs from religious traditions have been found to be associated with appraisals, coping strategies, and stress-related outcomes (Newton & McIntosh, 2010).

### Challenge and Threat in the Stress Response

The biological response to stress originates from the cognitive interpretation (meaning assigned to the event), followed by the affective integration (blending the experienced emotion into the cognitive interpretation) and the resulting neurological triggering (activation of anatomical structures in the central nervous system responsible for the initiation of the stress response) (Everly & Lating, 2013). As such, anticipatory cognitive appraisals represent an important psychological mechanism associated with the potential to determine the magnitude, dynamics, and physiological profile of the stress response. In the context of psychological stressors, including the social-evaluative threat (SET), primary cognitive appraisals of challenge and threat have received the most attention. The appraisal of threat, characterized by the anticipated loss of social self-esteem and rejection, is clearly distinguished from the appraisal of challenge, which relates to recognizing the potential for gain or growth in stressful situations (Folkman & Lazarus, 1984). Empirical investigations demonstrated that in the context of SET, primary cognitive appraisals robustly predicted the activation of the major stress system, the hypothalamic–pituitary–adrenal (HPA) axis (Gaab et al., 2005). Furthermore, primary cognitive appraisal assessed before stress affected the heart rate (Mayor & Gamaiunova, 2014).

The idea that cognitive evaluations determine not only the magnitude but also the particularity of stress-related physiological changes has been based on the BPS model of challenge and threat (Blascovich & Tomaka, 1996; Tomaka et al., 1993). In the framework of the BPS model, challenge and threat deviate from definitions proposed in earlier works (Folkman & Lazarus, 1984) and are conceptualized in terms of the motivation states of approach (challenge) and withdrawal (threat). Specifically, motivated performance gives rise to a state of challenge if the evaluated resources are equal to or greater than the demands. In contrast, the state of threat arises when demands are more significant than resources (Blascovich & Tomaka, 1996). These two states can be differentiated by cardiovascular measures, especially the cardiac output (CO), which reflects the amount of blood pumped from the heart each minute, and the total peripheral resistance (TPR), a measure of the resistance to blood flow throughout the circulatory system. During the state of challenge, arteries dilate, resulting in relatively higher CO and lower TPR than during threat, when the constriction of arteries results in less blood being pumped from the heart (Seery, 2011). The physiological pattern associated with challenge represents a more adaptive stress response, as it enables the response to metabolic demands to occur speedily (Tomaka et al., 1993); conversely, increased vascular resistance, which characterizes the threat response, impedes the delivery of oxygenated blood to the periphery and brain. The state of threat has significant health implications, straining the immune and cardiovascular systems due to increased arterial constriction (Blascovich, 2008), and is theorized to affect cellular aging (Epel et al., 2009).

Both the Transactional Theory of Stress and Coping (Folkman & Lazarus, 1984) and the BPS model (Blascovich & Tomaka, 1996; Tomaka et al., 1993) deal with the judgment process of stressful events and distinguish challenge and threat as potential evaluative stances. Both theories have been used in several recent empirical studies. The insights from the BPS model laid the foundation for the stress studies on blunted cardiovascular reactivity (Hase et al., 2020), flow theory (Scheepers & Keller, 2022), and music performance (Guyon et al., 2020), among other subjects. The Transactional Theory of Stress and Coping was used, e.g., in stress-related studies on social media (Wolfers & Utz, 2022), life satisfaction (Milas et al., 2021), and teachers’ stress (Herman et al., 2020). However, as described above, the two theories have significant conceptual differences and, consequently, different implications for the psychophysiology of the stress response. In the BPS model, challenge and threat constitute end states and are determined by the perceived demand/resources ratio, whereas in the transactional model, challenge and threat refer to perceived potentials for gain and loss. Combining insights from both theories allows for a more comprehensive assessment of the cognitive appraisal process in the context of social stress.

### Mindfulness-Based Interventions

Contemplative practice (CP) represents a form of training focused on developing awareness, concentration, wisdom, and regulatory abilities, among other skills, with the aim of profound psychological transformation (Davidson & Dahl, 2017). MBI is an umbrella term for behavioral programs based on contemplative practices originating in various religious and spiritual traditions (primarily rooted in Buddhism). MBIs come in several forms and focus mainly on cultivating mindfulness, broadly defined as openly attending to the present-moment experience with awareness (Creswell, 2017). Mindfulness stress reduction program (MBSR) (Kabat-Zinn, 2013) represents one of the earliest MBIs introduced in clinical practice. While based on Buddhist practices, MBSR is secular and does not explicitly refer to Buddhism. Such references only appear in SG-MBIs, which explicitly add several elements of Buddhist contemplative disciplines, including the cultivation of ethical and empathic awareness (loving-kindness and compassion meditation) and development of wisdom (e.g., analytical types of meditation) (Van Gordon & Shonin, 2020).

As suggested by the MBSR acronym, stress reduction was a crucial health-related outcome targeted by the earliest MBIs (Kabat-Zinn, 2013). Even though research results are still inconclusive, current empirical evidence suggests that MBIs help to reduce the stress response to psychological stressors with social-evaluative components, with effects observed for the immune, cardiovascular, and neuroendocrine systems (Morton et al., 2020). Program specificity plays an important role, with certain types of MBIs being more efficient than others (Engert et al., 2017; Lindsay et al., 2018). Several theoretical frameworks have been proposed to delineate the neurobiological mechanisms of MBIs and CP (Tang et al., 2015; Vago & David, 2012). The mindfulness stress-buffering account (Creswell & Lindsay, 2014) focused primarily on the stress-attenuating effects of MBIs. It proposed to differentiate the “top-down” regulation, where the stress reduction is initiated by activating the prefrontal regions of the brain, from the “bottom-up” regulation, which reduces stress reactivity by acting on the peripheral nervous system.

The neurobiological mechanisms of MBIs are tightly intertwined with psychological mechanisms. Emotion regulation strategies, especially acceptance and reappraisal, have been studied mainly as potential mechanisms for the stress-buffering effects of MBIs (Gamaiunova et al., 2019; Garland et al., 2011; Lindsay et al., 2018). Anticipatory cognitive appraisals, on the other hand, have received much less attention and have not been sufficiently tested as potential mechanisms for the effects of MBIs. This is unfortunate since, at least at the theoretical level, several components of contemplative training may impact the cognitive evaluation of a stressful situation.

First, MBIs of all types foster the development of decentering, which is a capacity to shift the experiential perspective from within onto that experience (Bernstein et al., 2015). Distancing from internal experience and the ability to observe the contents of thoughts can lead to a different primary appraisal or facilitate rapid reappraisal (Astin, 1997; Bernstein et al., 2015). Second, MBIs, and to a more significant degree, SG-MBIs, cultivate the development of compassion, self-compassion, and a benevolent attitude toward others, which are theorized to reduce threat perception through the development of the sense of self-worth independent of external evaluation or approval (Neff & Vonk, 2009). On top of that, they reduce proneness to self-conscious cognitions such as self-criticism (Gilbert & Procter, 2006). These changes to the relationship mode of stressful transactions may help to minimize threat perception in subsequent stressful encounters. Third, SG-MBIs, which include philosophical components in training (in the form of discourses, analytical meditations, etc.), can foster the creation of a cognitive schema—a mental representation that includes organized knowledge and the relational configuration of a particular domain (Van Gordon & Shonin, 2020). This cognitive lens impacts the appraisal of a stressful event (McIntosh, 1995; Newton & McIntosh, 2010). For example, the traditional Buddhist notion of non-self (i.e., anattā) leads to the understanding that all phenomena, including the self, do not possess inherent existence (Van Gordon et al., 2017). The interiorization of this notion can lead to changes in self-concept, where the self is seen as a mental construct, and less effort is subsequently mobilized for its protection or enhancement (Ryan & Brown, 2003). In the context of SET, it might be an essential factor for changes in threat evaluation, where the distress is primarily generated by the fear of losing a positive self-image.

As such, reducing self-concern through MBIs represents a potent mechanism of stress-attenuating effects. At the empirical level, worldviews from religious traditions were previously found to impact physiological reactivity to stress (Schnell et al., 2020), suggesting that religious beliefs might influence the cognitive appraisal process (Koenig & Cohen, 2002). Religious stimuli were also found to influence cardiovascular responses to motivated-performance situations (Weisbuch-Remington et al., 2005), suggesting that the challenge/threat cardiovascular profile in stress response is affected by elements of a religious system. In summary, contemplative training can alter the primary evaluation of psychological stressors by several possible mechanisms.

Only a few empirical studies investigated the relationship between contemplative training and primary cognitive appraisals of challenge and threat. The results are relatively heterogeneous. In a study investigating the psychological mechanisms of long-term meditation practice and stress response, no significant association was found between contemplative training and anticipatory appraisals of challenge and threat (Gamaiunova et al., 2019). On the other hand, in a longitudinal study, mindfulness was associated with reduced threat appraisal (Weinstein et al., 2009). In the framework of the BPS model of challenge and threat, the effects of MBI on cardiovascular profiles during social stress have been investigated in a randomized controlled trial of a mindfulness-based weight loss intervention (Daubenmier et al., 2019). The results suggested that mindfulness training increased challenge-related appraisals and resulted in cardiovascular reactivity associated with challenge. Another study investigated the effects of awareness manipulation and a brief acceptance training on cardiovascular stress responses (social-evaluative cold pressor test) underlying challenge and threat (Manigault et al., 2021). The results demonstrated that the combination of enhanced awareness and acceptance training was significantly associated with a higher CO and lower TPR, indicating greater challenge and lesser threat.

Given the scarcity of the above-mentioned research, further investigation is needed. In particular, it is necessary to establish whether MBIs *affect* the cognitive evaluation of social stressors and whether specific types of MBIs, such as SG-MBIs, produce more significant effects. Both the causal link between various forms of contemplative training and psychophysiological correlates, as well as the add-on effects of SG-MBIs, can be explored with randomized control experiments. This design allows for establishing a causal link between various forms of contemplative training and psychophysiological correlates of cognitive appraisal and testing the add-on effects of SC-MBIs experimentally.

This study presents a secondary analysis of a randomized controlled experiment investigating the effects of two MBIs on the psychophysiological response to social-evaluative stress (Gamaiunova et al., 2022). The two MBIs are the standard MBSR and MBSR-B, a SG-MBI with additional modules based on other Buddhist practices. Both were evaluated after an eight-week intervention. The stress response was measured across different physiological systems related to stress: the HPA axis and the autonomic nervous system (ANS), which is a component of the peripheral nervous system involved in the regulation of involuntary physiological processes, such as heart rate, blood pressure, and respiration. The primary analysis results demonstrate that MBIs reduce the magnitude of stress response in several physiological systems, with slightly higher effects seen in the case of MSBR-B.

The secondary analysis performed in this study explores the effects of MBIs on cognitive appraisals and associated cardiovascular profiles. The first aim is to test whether MBIs affect anticipatory cognitive appraisals of challenge and threat, measured by self-report. We hypothesized that MBI groups would show lower threat appraisal scores and higher challenge appraisal scores, with a more significant effect seen for MBSR-B compared with the control group. The second aim is to test whether the physiological profiles of the MBI groups correspond to challenge appraisal (an increase in CO and a decrease in TPR). We hypothesized that MBI groups would exhibit physiological profiles associated with challenge.

## Methods

### Participants

Participants were recruited in the Lausanne region and the university campus of Lausanne via flyers, online advertisements in a local paper, and a promotional website. The same set of participants was used for the primary- and secondary-analysis studies. The optimal total sample size of 72 participants (effect value of *f* = 0.4, with a significance level set at *α* = 0.05, power 1 − *β* = 0.85) was determined before recruitment using the G-Power software (Faul et al., 2007). The inclusion criteria were as follows: age 18–40 years; no prior regular practice of meditation (more than three hours per week); a good mastery of the French language; and the ability and desire to participate in the group sessions, do home assignments and participate in a one-day retreat. The exclusion criteria were prior participation in the TSST, chronic or acute mental or physical disease, addiction to substances, use of medications that interfere with the HPA axis or ANS functioning, severe obesity (Body Mass Index > 30), smoking more than five cigarettes per day, pregnancy or lactation, and inability to give consent. Out of 182 interested individuals, 52 did not meet the inclusion criteria, 31 declined to participate, and a sample of 99 participants was randomized into the three experimental groups. Due to attrition, a sample of 65 participants [MBSR (*n* = 20), MBSR-B (*n* = 21), Control (*n* = 24)] was included in the analysis of self-report data and 62 in the analysis of the physiological assessments (for the CONSORT flow diagram, see Gamaiunova et al., 2022). Individual characteristics of the participants are presented in Table A1 (Supplementary Materials).

**Table 1:** Descriptive Statistics and Group Differences in Outcome Variables

Outcome	Group descriptives *M* (SD)			ANOVA	Contrasts *p*^a^*, (*Cohen’s *d)*	
	CNTR (*N* = 24)	MBSR (*N* = 17)	MBSR-B (*N* = 21)		CNTR vs. MBSR	CNTR vs MBSR-B
Cardiac output (l/min)						
Rest	4.65 (1.94)	3.80 (1.02)	3.82 (1.48)			
Mid-task	4.95 (2.12)	3.94 (1.10)	4.33 (2.15)			
Reactivity (Δ)	0.30 (1.35)	0.15 (0.78)	0.50 (1.07)	*F*(2, 59) = 0.503, *p* = .697, η2_G_ = .02		
Total peripheral resistance (dyn·s/cm^−5^)						
Rest	1667.73 (731.85)	1724.32 (461.25)	1911.99 (695.62)			
Mid-task	1741.54 (727.71)	1851.02 (491.09)	1930.14 (745.04)			
Reactivity (Δ)	73.81 (466.95)	126.70 (354.03)	18.15 (388.79)	*F*(2, 58) = 0.758, *p* = .473, η2_G_ = 0.03		
Anticipatory cognitive appraisals (PASA)						
Challenge	3.35 (0.92)	4.12 (1.20)	4.20 (0.55)	*F*(2, 59) = 5.921*, p* = .01, η2_G_ = .17	*p* = .03(0.71)	*p* = .007(1.12)
Threat	3.62 (1.31)	3.57 (0.93)	3.19 (0.99)	*F*(2, 59) = .934, *p* = .399, η2_G_ = .03		
In-group differences between challenge and threat	Welsh t test *p (Cohen’s d*)					
	*p* = .43, *d *(− 0.23)	*p* = .151, *(*0.51)	*p* < .001 (1.26)			

CNTR = control group, MBSR = Mindfulness-Based Stress Reduction, MBSR-B = modified Mindfulness-Based Stress Reduction, PASA = Primary Appraisal Secondary Appraisal (Gaab et al., 2005)

^a^Results of Tukey’s HSD post hoc tests

### Procedure

Eligible participants were scheduled for the first visit, during which they received additional details on their participation in the study, signed the informed consent form, received a subject ID (assigned sequentially), and were randomized into one of the three conditions, stratifying for sex. The enrolled participants were blinded to their study conditions. After eight weeks of intervention (or the wait in the wait-list control condition), participants were scheduled to undergo a laboratory TSST session. They were asked to avoid caffeine, alcohol, food, and strenuous exercise two hours before the session. On arrival, participants underwent a pre-experimental check by answering questions on their current mood, sleep during the previous night, and medication. After that, they were connected to the physiological recording device (see “Materials”).

For social stress manipulation, we modified the TSST: the anticipation period was increased to 15 min to assess the pre-performance stress reactivity for the primary analysis (Gamaiunova et al., 2022). Two confederates dressed in white coats and with clipboards entered the room and presented the task. Then, the participant was instructed to complete a questionnaire to assess the cognitive evaluation of the upcoming task (see Materials) and was given 15 min to prepare. After the preparation, the participant delivered a five-minute speech and performed a five-minute arithmetic task in front of the evaluators, who maintained a critical attitude and used a camera. After the task, the participant was asked to complete questionnaires (not presented here) and remained attached to the physiological device for 30 min. The data were collected as follows: self-reported cognitive appraisals were assessed after the task introduction; physiological data were collected continuously for the impedance measures (the experimenter introduced time stamps during the procedure to mark the beginning and the end of corresponding parts of the experiment) and periodically for blood pressure (during rest and in the middle of the task periods). In addition, we collected six saliva samples (not presented here). The local Ethics Committee approved the study, and all participants signed a-priory and a-posteriori consent forms and were fully debriefed after the participation.

## Materials

### Intervention

Participants were randomly assigned to MBSR, MBSR-B, and wait-list control. MBSR was a standard protocol administered by a certified instructor comprising seven weekly group sessions (two hours each), a retreat day during week seven, and home practice for 55 min a day (Kabat-Zinn, 2013).

The MBSR-B program was designed based on MBSR in collaboration with the MBSR instructor and advanced meditation practitioners in Buddhist traditions. The MBSR-B program followed the same outline as the standard MBSR. In addition, each week focused on a particular concept from a broader Buddhist practice: week one concentrated on impermanence, week two on ethical aspects, week three on loving-kindness, week four on compassion, week five on the notion of not-self, week six on craving, and week seven on a choice of topics introduced earlier. The introduction of the concept included a short discourse administered during the group session, audio instructions on how to apply the teachings informally in daily life, and guided meditation on the topic that should be listened to at home. For example, for the “not-self” topic, participants were asked to be aware of the moments of “selfing” (moments of increased sense of agency, heightened self-conscious emotions, moments of excessive preoccupation with one’s social image, etc.) during the week versus the moments of mindful activities (for details, see Supplementary Materials, Table A2).

### Measures

#### Cardiovascular Measures

Electrocardiography and impedance cardiography data were continuously collected using the Bionex data acquisition unit (MindWare Technologies, Gahanna, OH) with a sampling rate of 1000 Hertz (Hz). We placed seven spot electrodes on the participant’s thorax (Sherwood et al., 1990) and recorded the measured values using the BioLab acquisition software. The raw data file from the acquisition software was imported to the analysis software IMP 3.1.6 (MindWare Technologies, Gahanna, OH, USA). The distance between the front electrodes was introduced to the analysis software manually.

Each data segment was inspected online for the artifacts by a trained researcher, and the summary of values was exported to the data file. Blood pressure was assessed with a digital automatic blood pressure monitor Omron HEM-907 (Vernon Hills, Illinois, USA), which determines blood pressure by oscillometric measurement. The device has been assessed previously for accuracy and has passed clinical evaluation (White & Anwar, 2001). Blood pressure was measured periodically: three times at the end of the rest period and three times in the middle of the task, and the values were recorded in the data file. The stroke volume (SV) was calculated offline using the Bernstein equation (Bernstein & Lemmens, 2005). The CO was calculated as SV × HR, where HR = heart rate, while the TPR was calculated as CO/MAP*80, where MAP = (DBP) + (SBP − DBP)/3 with MAP standing for mean arterial pressure, DBP for diastolic blood pressure, and SBP for systolic blood pressure.

#### Data Reduction

Continuous impedance signals were ensemble-averaged using one-minute epochs, and the scores were assessed as follows: the last two minutes of the rest period for rest and one minute in the middle of the task (mid-task). The blood pressure measurements were averaged as follows: three consecutive measurements for rest and three consecutive measurements for mid-task.

#### Self-report Measures

The transactional stress questionnaire or Primary Appraisal Secondary Appraisal, PASA (Gaab et al., 2005) is a 16-item questionnaire that measures the primary cognitive appraisals of threat and challenge and secondary appraisals (control expectancy and self-concept of one’s abilities). This questionnaire allows for assessing the cognitive evaluation of an upcoming stressful event and is widely used, especially in laboratory tasks with stress-inducing paradigms. Only primary appraisals (threat and challenge) subscales were reported in this study. The threat and challenge subscales consist of four items, with response options ranging from one (completely disagree) to six (completely agree). Higher scores indicated higher anticipatory challenge or threat. The value of Cronbach’s alpha was 0.79 for threat and 0.80 for challenge.

### Analyses

Statistical analyses were performed using R Studio version 1.3.1093. Missing values (7.8% in the physiological variables) were treated with the predictive mean matching (PMM) single imputation method using R package MICE, version 3.9.0 (van Buuren & Groothuis-Oudshoorn, 2011). Extreme outliers were identified as values higher than Q3 + 3 × IQR or below Q1 − 3 × IQR (where IQR corresponds to the interquartile range, Q1 to the first quartile, and Q3 to the third quartile) and removed from the dataset.

#### Self-report

As the scores of challenge and threat were not significantly correlated (*r* = 0.14, *p* = 0.29), we tested for group differences in scores of challenge and threat with two separate univariate analyses of variance (ANOVA), adjusting for multiple testing with the Holm–Bonferroni approach (Holm, 1979). A significant result from the ANOVA test was followed by Tukey’s honest significant difference post hoc test. Additionally, we tested for the difference between challenge and threat scores in each group using separate Student’s t tests. Before performing ANOVA, the following assumptions were made: absence of significant univariate outliers, normality, multicollinearity, linearity, homogeneity of variance–covariance, and homogeneity of variance. Before performing the Student’s t tests, the following assumptions were made: normality, homogeneity of variance, and extreme outliers. The reported effect size indices included generalized eta squared for ANOVA and Cohen’s *d* for t tests.

#### Physiological Variables

Physiological variables task engagement and the associated sympathetic activation are prerequisites for examining the CO and TPR as challenge and threat markers. We first tested whether TSST evoked changes in the pre-ejection period (PEP), an index of sympathetic activation, in all groups. To test group differences in the changes in CO and TPR, we computed reactivity values (delta), representing the difference between task performance and the pre-stress rest period (Llabre et al., 1991). Further, we tested for group differences in the delta values of CO and TPR using two separate univariate ANOVAs.

## Results

### Preliminary Analysis

The preliminary analysis results are reported elsewhere (Gamaiunova et al., 2022). A summary of the results can be presented as follows. The groups did not differ significantly in age, sex, education, occupation, marital status, or income. Concerning the practice of the MBI conditions, the two groups (MBSR and MBSR-B) were similar regarding the duration of practice (in minutes) in the course or self-reported difficulty and effort. The pre-experimental check did not show significant differences between the groups in the number of hours of sleep, perceived sleep quality, and mood.

### Main Analysis

#### Anticipatory Cognitive Appraisal (Self-report)

We performed two univariate ANOVAs to test for group differences in anticipatory cognitive appraisals of challenge and threat. The inspection of the QQ plots suggested the normal distribution of the data, and the results of Levine’s test indicated no significant difference between variances across groups. No significant group differences were observed for the threat scores: *F*(2, 59) = 0.934, *p* = 0.399, *η*2_G_ = 0.03 (Table 1). However, the groups differed in the challenge scores: *F*(2, 59) = 5.921*, p* = 0.01, *η*2_G_ = 0.17. The MBSR group had a higher challenge score than the control group (*p* = 0.03, Cohen’s *d* = 0.71). A significant difference, with a larger effect size, was also observed between the MBSR-B and control groups (*p* = 0.007, Cohen’s *d* = 1.12). No statistically significant differences were found between the meditation groups (Table 1).

**Fig. 1:**
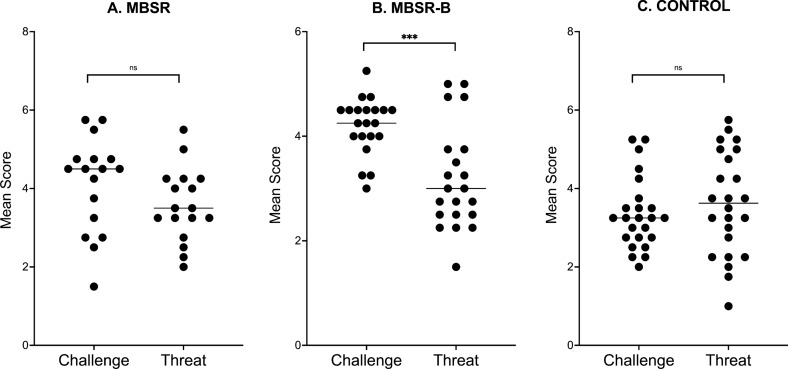
Differences in the Anticipatory Cognitive Appraisals of Challenge and Threat in the Treatment Groups. CONTROL = control group, MBSR = Mindfulness-Based Stress Reduction group, MBSR-B = modified Mindfulness-Based Stress Reduction group

As an exploratory analysis, we compared the scores of challenge and threat in each group. The homogeneity of variance assumption was violated for the two groups, and we performed Welsh t tests. No statistically significant difference was found between challenge and threat in the MBSR group (*t*(30.16) = 1.473, *p* = 0.151, Cohen’s *d* = 0.51) or control group (*t*(41.18) = − 0.797, *p* = 0.43, Cohen’s *d* = − 0.23). However, the challenge and threat scores differed significantly in the MBSR-B group (*t*(31.06) = 4.091, *p* < 0.001, Cohen’s *d* = 1.26) (Fig. 1).

#### Task-Related Sympathetic Activation

To check if all three groups demonstrated sympathetic activation due to the task, we performed repeated-measures ANOVA for each group, testing for PEP changes from rest to mid-task. PEP significantly decreased in the MBSR group from rest (*M* = 104.50, SD = 12.21) to mid-task (*M* = 99.59, SD = 15.36; *F*(1, 16) = 5.378, *p* = 0.034, η2_G_ = 0.03); MBSR-B group from rest (*M* = 109.98, SD = 2.40) to mid-task (*M* = 97.91, SD = 12.97; *F*(1, 20) = 37.115, *p* < 0.001, η2_G_ = 0.18); and control group from rest (*M* = 106.19, SD = 13.57) to mid-task (*M* = 90.08, SD = 14.24; *F*(1, 23) = 39.476, *p* < 0.001, η2_G_ = 0.26). These results (increased ventricular contractility) suggest sympathetic activation in all three groups, allowing further analyses of challenge and threat cardiovascular profiles.

#### Cardiac Output and Total Peripheral Resistance

**Fig. 2:**
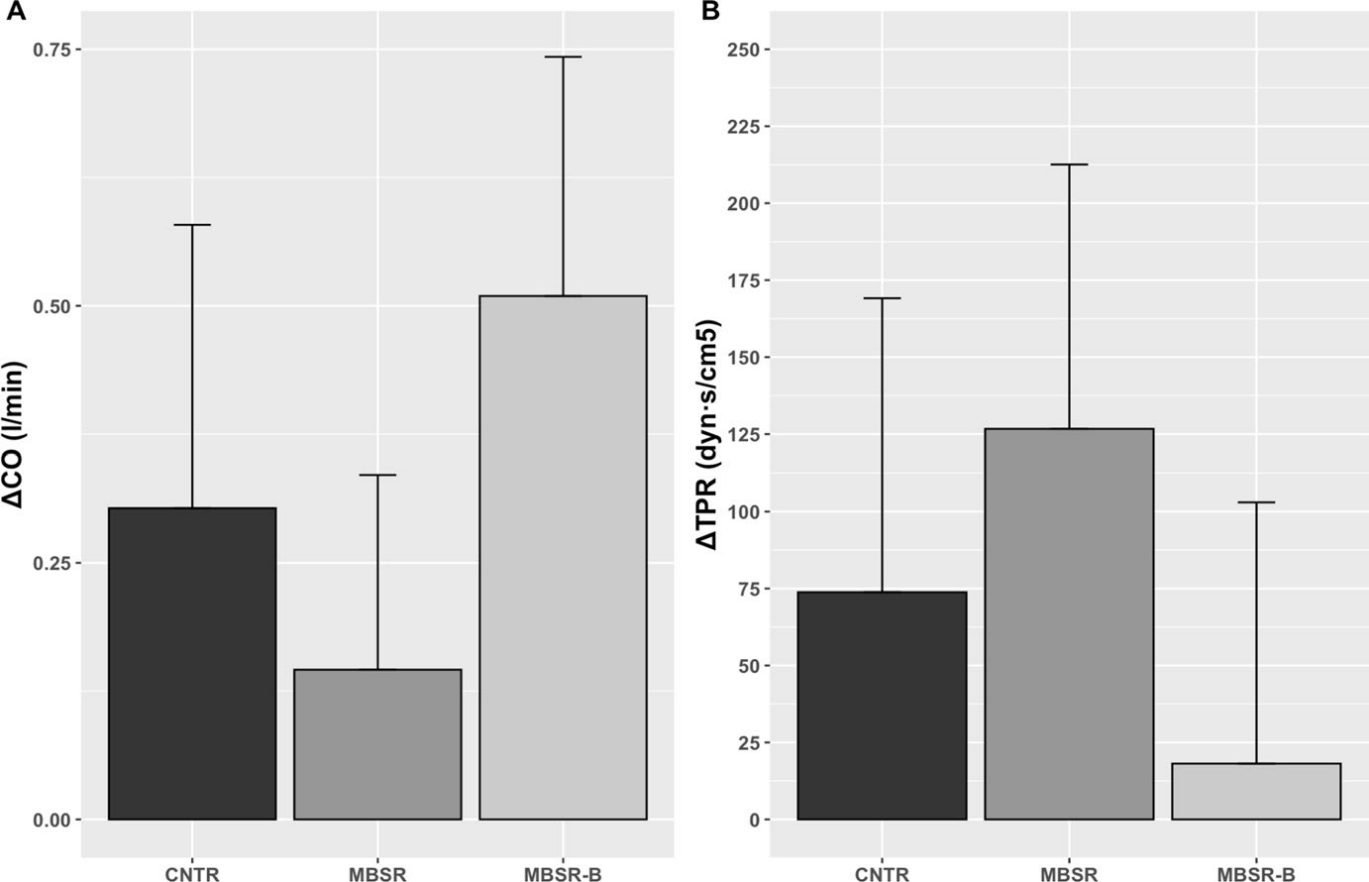
Changes (Delta) in Cardiac Output and Total Peripheral Resistance. Note. CNTR = control group, MBSR = Mindfulness-Based Stress Reduction group, MBSR-B = modified Mindfulness-Based Stress Reduction group

To test for group differences in the changes in CO and TPR, we performed univariate ANOVA on the reactivity values (delta), representing the difference between task performance and pre-stress rest. The inspection of the QQ plots suggested the normal distribution of the data, and the results of Levine’s test indicated no significant difference between variances across groups. The groups did not show statistically significant differences in either CO reactivity (*F*(2, 59) = 0.503, *p* = 0.697, η2_G_ = 0.02) or TPR reactivity (*F*(2, 58) = 0.758, *p* = 0.473, η2_G_ = 0.03) (Fig. 2). We performed exploratory analyses and tested for CO and TPR changes from rest to mid-task in each group. The results revealed that CO showed a statistically significant increase in the MBSR-B group (*F*(1, 20) = 4.781, *p* = 0.04, η2_G_ = 0.02) but not in MBSR (*F*(1, 16) = 0.591, *p* = 0.453, η2_G_ = 0.01) or control (*F*(1, 23) = 1.212, *p* = 0.282, η2_G_ = 0.01) groups (Table 1 shows the descriptive characteristics).

## Discussion

In this study, we investigated the effects of two MBIs on the (1) self-reported anticipatory cognitive appraisals of challenge and threat (Folkman & Lazarus, 1984) before a social-evaluative stress task, and (2) cardiovascular profiles of challenge and threat in the framework of the BPS model (Tomaka et al., 1993). Our findings suggest that MBSR-B is associated with higher in-group challenge scores and shows a more pronounced cardiovascular profile of a challenge as per the BPS model. We offer a potential explanation for our findings, address the study limitations, and propose future directions for research.

The analysis of self-reported data demonstrates that the threat levels experienced by the participants before the task did not differ significantly among the experimental groups. However, both meditation groups scored higher than the control group on challenge appraisal. Further, comparing the challenge and threat levels in each group reveals that one of the MBI groups (MBSR-B) scores significantly higher for the challenge than for the threat. In the transactional model of the stress and coping theory (Lazarus & Folkman, 1987), challenge and threat are not conceptualized as mutually exclusive. They can co-occur when the event is judged in its potential to bring harm/loss and mastery/gain simultaneously. Our results suggest that while all three groups show mixed appraisals for challenge and threat, the MBSR-B group’s anticipatory appraisal has a more substantial challenge component. From a psychophysiological standpoint, these findings can explain heterogeneous research results in studies evaluating the effects of MBIs on stress-related changes in the ANS (Morton et al., 2020). Challenge is characterized by motivational engagement and certain positive emotions, such as joy, which are associated with increased beta-adrenergic sympathetic activation (Kreibig, 2010). This may explain why the effects of MBIs on the ANS stress response attenuation have been found in certain studies (Nyklíček et al., 2013) but not in others (Creswell & Lindsay, 2014; Engert et al., 2017).

Regarding the level of physiological activation in response to stress, none of the groups shows a clear physiological profile associated with the challenge rather than the threat as conceptualized by the BPS model: participants in all three groups demonstrated an increase in CO and, to a minor degree, in TPR. However, the challenge pattern was more pronounced in the MBSR-B group: the exploratory analysis demonstrates that only this group exhibits the statistically significant increase in the CO from rest to task. One might hypothesize that two other groups show a bivalent activation of appraisals, although additional physiological indices are necessary to confirm this hypothesis (Uphill et al., 2019). It is important to note that in the framework of the BPS model, challenge and threat are considered end states and are primarily determined by the perceived demand/resources ratio. This differs from the transactional model, where challenge and threat refer to perceived potentials for gain and loss, respectively, and are determined by physiological activation (Seery, 2011). Even though challenge is conceptualized differently in these models, MBSR-B is associated with both.

The question of program specificity has been raised previously, and it was shown that different types of contemplative training do not impact stress response similarly (Engert et al., 2017; Morton et al., 2020). In line with these findings, this study suggests that only the MBSR-B program scores higher on challenge than threat on the level of self-report. Moreover, only the MBSR-B induces the cardiovascular profile, which is more challenge-like than threat-like. Two types of practices in the MBSR-B program could be responsible for promoting challenge rather than threat-oriented cognitive appraisals. First, the additional module of MBSR-B contains practices aimed at understanding Buddhist concepts of not-self, the origin of suffering, and impermanence, followed by their application to stressful encounters. The possible engagement of these concepts during a stressful encounter represents a form of religious coping in which elements of a traditional doctrine form a cognitive lens (McIntosh, 1995) through which the stressful encounter can be viewed. Buddhist philosophical tenets allow perceiving experiences as fleeting and independent of the existing self, serving as an essential antecedent of the cognitive appraisal process (Folkman & Lazarus, 1984). In stress research, this notion is similar to social safety schemas about the self and social world, theorized to profoundly impact physiological stress responses via cognitive evaluation processes (Slavich, 2020). Second, the module also includes practices involving the development of compassion (focus on the awareness of others’ suffering) and loving-kindness (developing concern for the well-being of others). These practices impact interactional and interpersonal engagement (Hofmann et al., 2011) with the potential to promote social safety (Gilbert, 2009) and increase social connectedness and positivity toward strangers (Hutcherson et al., 2008), thus fostering motivational states of approach in social situations. A related skill of self-compassion is the stable feeling of self-worth that is not contingent on particular outcomes (Neff & Vonk, 2009); this represents a potential antecedent of the appraisal process. The results of an earlier empirical investigation suggested an association between self-compassion and the process of stress appraisal (Chishima et al., 2018). To conclude, several sources support our finding that practices introduced in the additional module of MBSR-B may provide additional stress-protective benefits.

### Study Limitations and Future Directions

This study has several limitations. First, the sample size was relatively small, as the study was powered for the primary analysis (Gamaiunova et al., 2022), and the initial sample underwent some attrition. We observed a statistically significant change in pre- to mid-task cardiac output only in the MBSR-B group. However, the difference in change score among the three groups was not detected. A larger sample size could help to detect the difference. Second, the additional module of MBSR-B combines practices from different meditation families. It is currently unclear which practices in particular contribute to more pronounced challenge appraisal observed in the MBSR-B group. Future studies on SG-MBIs and cognitive appraisals should address this question by comparing the effects of interventions from different families, including constructive, deconstructive, and attentional families (Dahl et al., 2015).

In this study, we focused only on primary appraisals. However, the consequences of the cognitive appraisal process on the stress response are twofold: first, primary appraisals directly influence the magnitude of physiological activation in response to stress (Gaab et al., 2005) and physiological response profile (Tomaka et al., 1993); second, primary appraisal processes affect the next step in the transactional process; that is, secondary cognitive appraisals and the choice of coping strategies (Folkman et al., 1986). Consequently, primary cognitive appraisals impact the magnitude of stress reactivity and the prolonged activation of the stress response due to a reduced sense of control or less efficient coping strategies. Future studies thus should address how MBIs affect secondary appraisals and the consequent choice of coping and emotion regulation strategies. This research direction is further supported by the results of a qualitative study on the experience of stress by meditation practitioners, which suggested that the practice was indeed associated with the meaning of the stressful event and the strategies chosen to deal with its consequences (Gamaiunova et al., 2021).

Several antecedents, such as beliefs, values, and goal hierarchies, determine the primary cognitive appraisal process. Therefore, another fruitful direction might be to explore the effects of contemplative approaches aimed at cultivating self-inquiry, spirituality, purpose, and meaning (Dahl & Davidson, 2019).

### Clinical Implications

Cognitive appraisals influence physiological reactivity during stress (Gaab et al., 2005; Maier et al., 2003) and partially mediate the relationship between stress and health outcomes (Gomes et al., 2016). Developing clinical interventions to modify cognitive evaluations of stressors represents a potential direction for behavioral approaches focused on stress reduction. Considering that specific beliefs or ideas from religious and spiritual frameworks influence the cognitive appraisal process (Newton & McIntosh, 2010), SG-MBIs might be particularly effective.

## Conclusion

To conclude, this study provides evidence of the causal effects of mindfulness-based contemplative training on the cognitive appraisal process, suggesting that MBIs promote challenge rather than threat appraisal of stressful events. Furthermore, the results suggest that MBSR-B, which includes additional practices from a larger Buddhist framework, might have a more significant effect on perceiving the stressful event as a challenge rather than a threat. These findings provide insights into psychological mechanisms underlying the effects of MBIs on stress and call for further elaboration and testing of the second-generation contemplative programs with elements of traditional practices and teachings.

## Supplementary Information

Below is the link to the electronic supplementary material.

Supplementary file1 (DOCX 26 KB)
