# Supplementary material for: Challenge or Threat? The Effects of the Standard and a Second-Generation Mindfulness Intervention with Buddhist Practices on Cognitive Appraisals of Stress: Secondary Analysis of a Randomized Controlled Experiment Performed in Switzerland
Source: J Relig Health. 2023 Dec 22;63(6):4773–92. doi: 10.1007/s10943-023-01964-8 (PMC11576654; doi:10.1007/s10943-023-01964-8)
Supplement: Supplementary file 1 — Supplementary file1 (DOCX 26 KB) [file 10943_2023_1964_MOESM1_ESM.docx]

**Supplementary materials for the article:**

**Challenge or threat? The effects of the standard and a second-generation mindfulness intervention with Buddhist practices on cognitive appraisals of stress: Secondary analysis of a randomized controlled experiment performed in Switzerland**

Liudmila Gamaiunova^a, b*^, Pierre-Yves Brandt^a^, Matthias Kliegel^b, c^

^*^ Corresponding author: liudmila.gamaiunova@unil.ch

^a^ Institute for Social Sciences of Religions (ISSR), University of Lausanne, CH-1015, Lausanne, Switzerland

^b^ Swiss National Center of Competences in Research LIVES–Overcoming vulnerability: life

course perspectives, Switzerland

^c^ Department of Psychology, University of Geneva, CH-1205, Geneva, Switzerland

**Table A1**

*Individual Characteristics of Participants*

| Individual characteristics | Frequency | | |
| --- | --- | --- | --- |
|  | MBSR  (*N* = 20) | MBSR-B  (*N* = 21) | WAITLIST  (*N* = 24) |
| Sex |  |  |  |
| males | 6 (30%) | 9 (43%) | 8 (33%) |
| females | 14 (70%) | 12 (57%) | 16 (67%) |
| Education |  |  |  |
| primary school | 0 | 0 | 0 |
| secondary school | 1 (5%) | 0 | 0 |
| professional school | 2 (10%) | 0 | 2 (8%) |
| gymnasium, pedagogical school, school of commerce | 1 (5%) | 2 (10%) | 3 (13%) |
| university, polytechnical school, HES | 15 (75%) | 16 (76%) | 18 (75%) |
| PhD or other post-grade university degree | 1 (5%) | 3 (14%) | 1 (4%) |
| Occupation |  |  |  |
| student | 4 (20%) | 8 (38%) | 12 (50%) |
| paid employment | 9 (45%) | 10 (48%) | 11 (46%) |
| self-employed | 6 (30%) | 1 (5%) | 1 (4%) |
| unemployed | 1 (5%) | 2 (9%) | 0 |
| retired | 0 | 0 | 0 |
| unable to work (disability) | 0 | 0 | 0 |
| Marital status |  |  |  |
| single | 10 (50%) | 6 (29%) | 12 (50%) |
| in a relationship | 6 (30%) | 11 (52%) | 9 (38%) |
| married | 4 (20%) | 4 (19%) | 2 (8%) |
| divorced | 0 | 0 | 1 (4%) |
| Income (CHF) |  |  |  |
| 0-39 999 | 13 (65%) | 12 (57%) | 12 (52%) |
| 40 000 – 79 999 | 4 (20%) | 6 (29%) | 7 (31%) |
| 80 000 – 100 000 and more | 3 (15%) | 3 (14%) | 4 (17%) |
| Prefer not to say | 0 | 0 | 1 |
| Prior meditation practice |  |  |  |
| 1 hr/week | 1 (5%) | 1 (5%) | 0 |
| 2 hrs/week | 1 (5%) | 0 | 2 (8%) |
| Age (years) | *M* (*SD*) | | |
|  | MBSR | MBSR-B | WAITLIST |
|  | 30.45 (6.14) | 28.67 (5.83) | 27.21 (4.56) |

**Table A2**

*Overview of the Additional Module Contents for the MBSR-B Program*

| **Week** | **Theme** | **10 minutes of introduction during class** | **10 minutes of audio instructions on how to apply it "informally" in daily life (day after class)** | **Practicing the informal practices when possible during the week** | **Daily 10-minutes guided meditation centered upon the topic** |
| --- | --- | --- | --- | --- | --- |
| 1 | impermanence | x | x | being aware of the impermanence of emotions (self and others) | - |
| 2 | Ethics | x | x | abstaining from one minor unethical action for the week (like gossiping), practicing one generous action | contemplation on gratefulness and generosity |
| 3 | Lovingkindness | x | x | using informal loving-kindness intentions during difficult conversations | lovingkindness meditation |
| 4 | Compassion | x | x | using informal compassionate intentions when meeting someone who faces difficulties | compassion meditation |
| 5 | Not-self | x | x | being aware of moments of "selfing" during the week vs moments of mindful activities | open awareness meditation with focus on "desidentification" |
| 6 | Craving | x | x | being mindful when craving is present, planning one pleasant activity that is not part of those that are associated with addiction (food, screens, etc.) | "urge surfing" meditation |
| 7 | No theme | - | - | any of the previously introduced practices | any of the previously practiced meditations |
